# EMRSA-15 Bacteremia is not Associated with a Worse Outcome Compared with Bacteremia Caused by Multidrug-Resistant MRSA

**Published:** 2007-06

**Authors:** Li-Yang Hsu, Nidhi Loomba-Chlebicka, Tse-Hsien Koh, Mei-Ling Kang, Ban-Hock Tan, Paul Ananth Tambyah

**Affiliations:** 1 *Department of Medicine, Yong Loo Lin School of Medicine, National University of Singapore, Singapore;*; 2 *Singapore General Hospital, Singapore*

**Keywords:** bacteremia, methicillin resistance, mortality, *Staphylococcus aureus*, outcomes

## Abstract

EMRSA-15 (ST22-MRSA-IV) is rapidly replacing the endemic ST239 health care-associated methicillin-resistant *Staphylococcus aureus* clone in Singapore. A one-year single-centre cohort study of inpatients with MRSA bacteremia was performed to determine if bacteremia caused by EMRSA-15 was associated with worse outcomes compared to bacteremia caused by the endemic ST239 strain. Strains were identified by antibiotypes, and subsequent validation was performed on a selected sample of MRSA strains via pulsed-field gel electrophoresis and staphylococcal chromosome cassette *mec* typing. Two hundred and twenty-eight patients with MRSA bacteremia were studied; Seventy-three were infected with EMRSA-15. EMRSA-15 and ST239-infected patients were similar regarding gender, frequencies of most co-morbidities, and risk factors for adverse outcomes. Similar numbers of EMRSA-15-infected and ST239-infected patients died (24.7% vs 27.1%, *P*=0.70) or developed complicated infections (41.1% vs 40.0%, *P*=0.88). After multivariate analysis, EMRSA-15 as a cause of bacteremia was not significantly associated with either death or development of complicated infections, although inappropriate therapy (5.45-fold, *P*<0.01) and a respiratory source of bacteremia (4.69, *P*<0.01) were independently associated with subsequent mortality. The increased propensity of EMRSA-15 for dissemination was not associated with increased virulence in our patients. Further work in determining the mechanisms by which highly transmissible MRSA spreads rapidly is required to better target infection control approaches at these important emerging MRSA clones.

## INTRODUCTION

Methicillin-resistant *Staphylococcus aureus* (MRSA) is a major public health concern in many countries. In addition to the high rates of mortality and morbidity associated with health care-associated MRSA (HA-MRSA) infections (1, 2), a significant financial and logistic burden is imposed on most healthcare systems in terms of preventive and/or treatment costs (2, 3).

Recent molecular work demonstrated that the majority of HA-MRSA strains worldwide arose from five *S. aureus* lineages (4). However, the status of different clones remains fluid, with multidrug-susceptible clones such as EMRSA-15, EMRSA-16, and the Berlin clone currently replacing older endemic multidrug-resistant HA-MRSA clones in many parts of the world (5). In Singapore, EMRSA-15 is rapidly replacing the endemic ST239 clone (6).

Few studies have compared the outcomes of infections caused by different HA-MRSA clones (7). Theoretically, dissimilar genetic backgrounds may give rise to differences in virulence and distinct clinico-epidemiologic presentations. Knowledge of such differences may enable resource-strapped institutions to focus assets on the containment/eradication of one particular clone, or permit the replacement of more virulent clones by MRSA that are susceptible to a greater array of antibiotics.

We performed a cohort study to determine if differences in outcomes existed between bacteremias caused by EMRSA-15 and ST239-MRSA-III, two major international clones belonging to different *S. aureus* lineages (CC22 and CC8 respectively) (4).

## METHODS

### Study design

A cohort study comparing the outcomes of inpatients with EMRSA-15 and ST239 MRSA bacteremia between 1 January and 31 December, 2005 at the Singapore General Hospital (SGH) – a 1,600-bed tertiary acute care hospital – was performed. The hospital’s ethics review board granted approval for the study.

### The cohort

Inpatients with one or more positive blood cultures for MRSA, and positive signs and symptoms of infection not attributable to other concurrent non-MRSA infections were evaluated.

MRSA strains were segregated by antibiotype: EMRSA-15-infected patients had bacteremias caused by strains that were susceptible to gentamicin, tetracycline and trimethoprim-sulfamethoxazole but were resistant to ciprofloxacin, erythromycin and clindamycin. ST239-infected patients had bacteremias caused by strains that were resistant to gentamicin, tetracycline, ciprofloxacin, erythromycin and clindamycin. We had shown that these respective anti-biotypes are exclusively restricted to EMRSA-15 and ST239 strains in SGH previously (6). Patients with MRSA possessing other antibiotypes were excluded.

### Chart review

Investigators blinded to MRSA antibiotypes reviewed the patients’ medical records, collating epidemiologic and clinical data in a designated database. The co-morbidites analyzed were: renal failure requiring dialysis at the point of bacteremia, diabetes mellitus, cardiovascular disease, valvular heart disease, cancer, exfoliative skin disease, recent surgery ≤28 days before bacteremia, and liver cirrhosis. Severity of illness was analyzed according to the McCabe-Jackson score (8).

Other factors potentially predisposing to adverse outcomes were analyzed: hypoalbuminemia (serum albumin <30 g/L) or anemia (hemoglobin <12 g/dL) at the point of bacteremia, steroid usage exceeding an equivalent of 10 mg of prednisolone/day for ≥one week prior to bacteremia, presence of implant (s), and inappropriate therapy. Inappropriate therapy was defined by any one of the following: failure to initiate anti-MRSA therapy within 24 hours of a positive blood culture result; suboptimal dosages of antibiotics (trough serum levels of <8 μg/ml were defined as suboptimal for vancomycin); antibiotic duration of <10 days for uncomplicated bacteremia and <28 days for endocarditis, bone, joint or implant infections; and failure to remove accessible foci of infection.

We used the diagnoses recorded by the attending physicians, with the following exceptions: pneumonia was defined by new pulmonary infiltrates on a chest radiograph and isolation of MRSA from purulent sputum; endocarditis was defined strictly according to Duke’s criteria (9); implants were considered infected only if positive cultures were obtained from the implants or surrounding tissue, or radiological signs of inflammation surrounding the implant were present; and urinary tract infection was defined by a leukocyte concentration of ≥50 leukocytes/mm^3^ of urine and a pure culture of MRSA yielding >10^5^ cfu/ml in patients with the appropriate clinical presentation.

Mortality – the primary outcome indicator – was attributed to MRSA if no other clear cause of death was present and signs of MRSA infection persisted. Complicated infection – defined as new infection at a site distant from the primary focus caused either by hematogenous seeding or direct extension of infection – was a secondary outcome measure.

### Molecular typing

To confirm our identification strategy, MRSA strains isolated from all clinical sites in May 2005 were collected. We confirmed the identity of *S. aureus* via colony morphology, coagulation of citrated rabbit plasma with EDTA (BBL Becton Dickinson and company, Cockeysville, MD, USA), and production of clumping factor and protein A (BactiStaph, Remel, Lenexa, KS, USA). Methicillin resistance was confirmed via typing of staphylococcal chromosome cassette *mec* (SCC*mec*) (10), and pulsed-field gel electrophoresis (PFGE) with *Sma*I macrorestriction was performed via previously described methods (11). Gel images were digitized and compared to stored results in our MRSA database using the Molecular Analyst v1.6 software.

### Statistics

Intercooled Stata (version 9.2) was used for statistical calculations. Dichotomous variables were analyzed with the χ^2^ test or Fisher’s exact test appropriately, and continuous variables were analyzed with Student’s *t* test. Univariate analyses of the association between individual variables and outcomes were performed using logistic regression. Variables with a *P* value of <0.2 on univariate analysis were included in the corresponding step-wise multivariate analyses. A *P* value of ≤0.05 was considered statistically significant.

## RESULTS

The records of all 228 patients who met study criteria were reviewed. Of these, 73 (32.0%) had EMRSA-15 and 155 (68.0%) had ST239 bacteremia based on antibiotypes. Eight other inpatients with MRSA bacteremia were excluded because of differing antibiotypes.

Descriptive characteristics of the cohort are shown in Table 1. Patients with EMRSA-15 bacteremia were slightly older than ST239-infected patients, but had similar frequencies of specific co-morbidities with the exception of a slightly higher proportion of cirrhotics. McCabe-Jackson severity scores at the time of bacteremia were similar.

**Table 1:** Descriptive characteristics for the cohort of patients with MRSA bacteremia

Characteristic	MRSA bacteremia		Significance (*p*-value)
	Cases – EMRSA-15 (n=73)	Controls – ST239 (n=155)	

Demographics:			
Male sex	37 (50.7)	96 (61.9)	0.11
Age, mean years (range)	68 (26-92)	64 (24-89)	<0.05
Co-morbidities:			
Renal failure on dialysis	44 (60.3)	97 (62.6)	0.74
Diabetes mellitus	45 (61.6)	75 (48.4)	0.06
Cardiovascular disease^a^	49 (67.1)	92 (59.4)	0.26
Valvular heart disease	2 (2.7)	11 (7.1)	0.19
Cancer^b^	18 (24.7)	38 (24.5)	0.98
Exfoliative skin disease	9 (12.3)	17 (11.0)	0.76
Recent surgery	20 (27.4)	61 (39.4)	0.08
Liver cirrhosis	6 (8.2)	4 (2.6)	0.05
Mean number of	2.68 (1-5)	2.71 (1-6)	0.82
co-morbidities (range)	-	-	-
McCabe score:			0.48
Fulminant	1 (1.4)	6 (3.9)	-
Ultimately fatal	54 (74.0)	115 (74.2)	-
Non-fatal	18 (24.7)	34 (21.9)	-
Other risk factors:			
Hypoalbuminemia	59 (80.8)	135 (87.1)	0.21
Anemia	64 (87.7)	139 (89.7)	0.65
Steroid use	6 (8.2)	21 (13.5)	0.25
Implants – all^c^	12 (16.4)	24 (15.5)	0.85
Orthopedic	7 (9.6)	12 (7.7)	0.64
Endovascular	3 (4.1)	9 (5.8)	0.59
Inappropriate therapy^d^	12 (19.0)	27 (18.9)	0.69
Presumed source of bacteremia:			
Catheter^e^	31 (42.5)	84 (54.2)	0.10
Respiratory tract	12 (16.4)	14 (9.1)	0.10
Wound	19 (26.0)	26 (16.7)	0.10
Urinary	3 (4.1)	4 (2.6)	0.53
Implant	1 (1.4)	4 (2.6)	0.56
Unknown	7 (9.6)	14 (9.1)	0.98
Outcomes:			
Complicated infection	30 (41.1)	62 (40.0)	0.88
Attributable mortality	18 (24.7)	42 (27.1)	0.70

Data are no. (%) of patients, unless otherwise indicated.

a Includes ischemic heart disease, congestive heart failure, and cerebrovascular events.

b Includes both solid organ and hematological malignancies.

c Orthopedic implants include joint replacements, plates & screws, hemiarthroplasties. Endovascular implants include cardiac valves, permanent pacemakers, aortic stents and arteriovenous grafts. Other implants are DJ stents and percutaneous nephrostomy tubes.

d Ten EMRSA-15 and 12 ST239 cases were excluded from analysis as they had died prior to knowledge of results of positive blood cultures.

e Includes central venous catheters, arterial lines, and peripheral venous cannulas.

Frequencies of risk factors, presumptive sources of bacteremia, and outcomes were similar for both groups. Overall attributable mortality was 26.3% (60 of 228), and complicated infections occurred in 40.4% of the cohort. Death occurred in 22 (9.6%) patients prior to identification of MRSA. Secondary sites of infection were evenly distributed among the cohort: pneumonia and/or empyema, 29 patients (12.7%); bone/joint infections, 27 patients (11.8%); urinary tract infections, 19 patients (8.3%); endocarditis/endovascular infections, 16 patients (7.0%); intra-abdominal infections, five patients (2.2%). There were two patients with pyomyositis, two with meningitis, and one with enophthalmitis. Seven patients had multiple secondary sites of infection.

For patients with MRSA identified prior to their demise, vancomycin monotherapy was used in 49 (77.8%) EMRSA-15-infected and 115 (80.4%) ST239-infected patients. Other regimens included linezolid, rifampicin and fusidic acid, and combinations of vancomycin with either rifampicin or gentamicin. Ten EMRSA-15-infected and 23 ST239-infected patients received inadequate dosages of vancomycin, while one per group received <10 days of therapy. Others classified as having received inappropriate therapy had unremoved foci of infection.

Results of univariate analysis for association of cohort characteristics with outcomes are shown in Table 2. Significant characteristics associated with mortality included age >65 years, cardiovascular and valvular heart diseases, inappropriate therapy, respiratory source of bacteremia, and recent surgery. Significant univariate predictors of complicated infection included age >65 years, lower McCabe-Jackson score, presence of implants, and respiratory or cutaneous sources of bacteremia.

**Table 2:** Univariate analysis of the impact of cohort characteristics on mortality and development of complicated infection

Characteristic	Attributable mortality		Complicated infection	
	Odds ratio (CI)	*p*-value	Odds ratio (CI)	*p*-value

Demographics:				
Male sex	1.61 (0.87-2.99)	0.13	1.50 (0.87-2.58)	0.15
Age >65 years	1.95 (1.05-3.62)	0.03	1.76 (1.02-3.02)	0.04
Co-morbidities:				
Renal failure on dialysis	1.20 (0.65-2.22)	0.56	0.59 (0.34-1.01)	0.06
Diabetes mellitus	1.25 (0.69-2.26)	0.47	1.20 (0.71-2.05)	0.49
Cardiovascular disease	2.25 (1.16-4.35)	0.02	1.49 (0.86-2.59)	0.16
Valvular heart disease	5.02 (1.57-16.00)	<0.01	1.78 (0.58-5.49)	0.31
Cancer	0.61 (0.29-1.29)	0.19	1.26 (0.69-2.32)	0.45
Exfoliative skin disease	1.17 (0.46-2.99)	0.74	1.87 (0.80-4.38)	0.15
Recent surgery	0.46 (0.23-0.90)	0.02	1.30 (0.75-2.25)	0.35
Liver cirrhosis	1.93 (0.53-7.08)	0.32	0.36 (0.07-1.7)	0.20
≥3 co-morbities	1.81 (0.82-4.00)	0.14	0.54 (0.26-1.13)	0.10
McCabe score:				
Fulminant	a	a	1.11 (0.24-5.09)	0.89
Ultimately fatal	1.06 (0.54-2.10)	0.86	0.42 (0.23-0.77)	0.05
Non-fatal	1.18 (0.59-2.35)	0.64	2.51 (1.33-4.71)	<0.01
Other risk factors:				
Hypoalbuminemia	1.80 (0.71-4.59)	0.22	1.11 (0.52-2.35)	0.79
Anemia	0.73 (0.30-1.80)	0.50	1.50 (0.62-3.64)	0.37
Steroid use	0.60 (0.22-1.67)	0.33	0.48 (0.19-1.18)	0.11
Implants-all	0.40 (0.15-1.09)	0.07	2.39 (1.16-4.92)	0.02
Orthopedic	0.47 (0.13-1.66)	0.24	1.92 (0.76-4.83)	0.17
Endovascular	0.54 (0.12-2.56)	0.44	1.51 (0.47-4.84)	0.49
Inappropriate therapy	6.11 (3.19-11.71)	<0.01	1.14 (0.63-2.06)	0.67
Presumed source of bacteremia:				
Catheter	0.56 (0.31-1.03)	0.06	0.19 (0.11-0.34)	<0.01
Respiratory tract	6.94 (2.89-16.66)	<0.01	7.86 (2.84-21.74)	<0.01
Skin/Soft tissue/Wound	1.02 (0.49-2.14)	0.95	5.05 (2.47-10.31)	<0.01
Urinary	a	a	3.85 (0.73-20.29)	0.11
Implant	a	a	0.99 (0.16-6.01)	0.99
Unknown	0.99 (0.39-2.52)	0.98	0.64 (0.26-1.58)	0.33
EMRSA-15 bacteremia	0.88 (0.46-1.67)	0.70	1.05 (0.59-1.84)	0.85

CI, 95% confidence interval.

a Numbers too small to calculate.

On multivariate analysis, only inadequate therapy (OR, 5.45; 95%CI, 2.64-11.25; *p*<0.01) and a respiratory source of bacteremia (OR, 4.69; 95%CI, 1.58-13.87; *p*<0.01) were independently associated with mortality. Independent predictors of complicated infection were respiratory (OR, 11.1; 95%CI, 1.64-74.89; *p*=0.01) and cutaneous sources of bacteremia (OR, 7.32; 95%CI, 1.38-38.88; *p*=0.02). EMRSA-15 as a cause of bacteremia was neither associated with mortality (OR, 0.63; 95%CI, 0.30-1.32; *p*=0.22) nor with complicated infection (OR, 0.67; 95%CI, 0.34-1.33; *p*=0.25).

**Figure 1:**
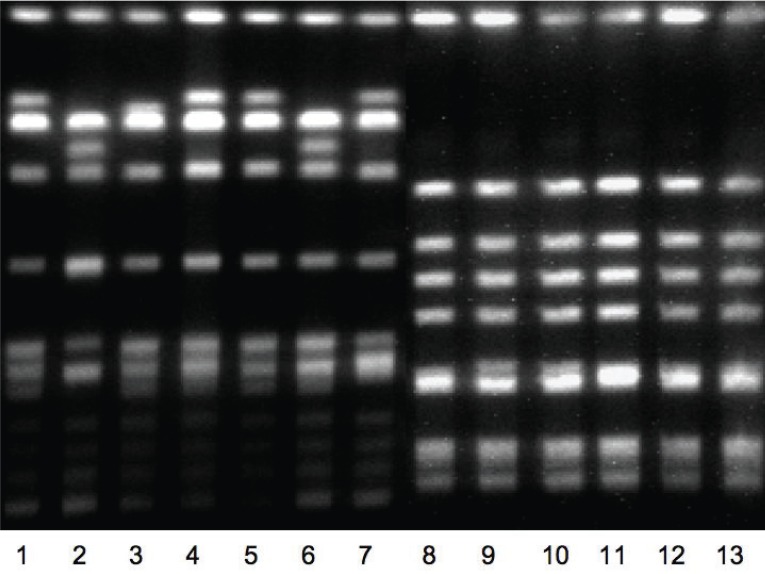
Representative gel image showing ST239 MRSA (lanes 1-7) and EMRSA-15 (lanes 8-13) on pulsed-field gel electrophoresis using *Sma*I restriction enzyme.

Ninety-five MRSA strains were isolated from clinical specimens in May. On PFGE and SCC*mec* typing, 61 (64.2%) strains were found to be ST239-MRSA-III and 32 (33.7%) were EMRSA-15 (ST22-MRSA-IV). A composite gel image displaying the PFGE patterns of these two clonal clusters is shown in Figure 1. Two strains (2.1%) were community-associated MRSA belonging to ST30-MRSA-IVc. The antibiotypes matched the study definitions, except for three EMRSA-15 strains that were resistant only to ciprofloxacin. While this profile matched the MRSA antibiotypes of four patients rejected during the selection process, these were not re-included because we no further verification could be obtained.

## DISCUSSION

This is the first study comparing the outcomes of infections caused by two major international HA-MRSA clones. There are several limitations, the most important being that bacteremia represents only a subset of all HA-MRSA infections. Using antibiotypes for identifying the two clones might have resulted in misclassification of MRSA, although the limited molecular typing performed suggested that misclassification bias, if present, was minimal. The sample sizes were also relatively small.

Nevertheless, there were pertinent findings. Our study showed no significant differences in mortality or risk of complicated infection between EMRSA-15 and ST239, suggesting that EMRSA-15 is not more pathogenic, despite its success in replacing ST239 in Singapore and elsewhere (6). In contrast to findings from Majorca (12), EMRSA-15 was also not significantly associated with bacteremia in our hospital – the percentage of EMRSA-15 in non-bacteremia cases caused by the two major MRSA clones (identified using antibiotypes) in SGH in 2005 was 31.6%.

Overall attributable mortality for MRSA bacteremia was comparable to previously published data (13). Our endocarditis rates were low possibly because only 72 (31.6%) patients underwent echocardiography, and of these, only 6 had trans-esophageal echocardiography. Nevertheless, there were no re-admissions for recurrent MRSA bacteremia during the period of study although delayed presentations might have been missed.

Despite susceptibility to a greater number of antibiotics, the treatment regimens used for EMRSA-15 in this study did not differ significantly from the regimens used for ST239. This is not surprising. Trimethoprim-sulfamethoxazole proved inferior to vancomycin in the only study comparing its use in MRSA (14), and other older drugs have either not been tested conclusively or have not demonstrated superiority to vancomycin for use in MRSA bacteremia.

In brief, our findings suggest that nosocomial bacteremia caused by the two epidemic HA-MRSA clones in Singapore did not differ with regards to the development of adverse outcomes. This complements the result of Seybold and co-workers, who showed that higher mortality rates did not occur even for cases of nosocomial bacteremia caused by the highly pathogenic USA300 community-associated MRSA (15). Nevertheless, the evidence is too weak at present to conclude that differing MRSA clones have no impact on the prognosis of noscomial MRSA bacteremia and infection.

Importantly however, the focus on the pathogenicity of different MRSA clones should not obscure the role of transmissibility in determining the overall virulence and healthcare burden of each particular clone. Higher transmission and colonization equates a higher number of infections, with attendant morbidity and mortality. A historical precedent has already been established in UK, where HA-MRSA infection rates increased dramatically after EMRSA-15 and -16 had replaced the majority of endemic clones in the 1990’s (16).

The rapidity of the spread of EMRSA-15 locally and elsewhere implies greater relative transmissibility compared to most other HA-MRSA clones. Other epidemic MRSA clones that are easily transmissible in the healthcare setting include EMRSA-16 (17, 18) and – perhaps rather counter-intuitively – USA300 (ST8-MRSA-IV), a community-associated MRSA clone (15, 19). This has long-term implications: it is unlikely that the spread of these clones can be contained by infection control measures that were unsuccessful against ST239 and other HA-MRSA clones. Attention needs to be directed at determining the mechanisms by which highly transmissible MRSA spreads rapidly to better target infection control approaches at these important emerging pathogens.
